# Preclinical *In vivo* Imaging for Fat Tissue Identification, Quantification, and Functional Characterization

**DOI:** 10.3389/fphar.2016.00336

**Published:** 2016-09-26

**Authors:** Pasquina Marzola, Federico Boschi, Francesco Moneta, Andrea Sbarbati, Carlo Zancanaro

**Affiliations:** ^1^Department of Computer Science, University of Verona, VeronaItaly; ^2^Preclinical Imaging Division – Bruker BioSpin, Bruker Italia s.r.l, MilanoItaly; ^3^Department of Neurosciences, Biomedicine and Movement Sciences, University of Verona, VeronaItaly

**Keywords:** fat, BAT, MRI imaging, CT imaging, PET imaging

## Abstract

Localization, differentiation, and quantitative assessment of fat tissues have always collected the interest of researchers. Nowadays, these topics are even more relevant as obesity (the excess of fat tissue) is considered a real pathology requiring in some cases pharmacological and surgical approaches. Several weight loss medications, acting either on the metabolism or on the central nervous system, are currently under preclinical or clinical investigation. Animal models of obesity have been developed and are widely used in pharmaceutical research. The assessment of candidate drugs in animal models requires non-invasive methods for longitudinal assessment of efficacy, the main outcome being the amount of body fat. Fat tissues can be either quantified in the entire animal or localized and measured in selected organs/regions of the body. Fat tissues are characterized by peculiar contrast in several imaging modalities as for example Magnetic Resonance Imaging (MRI) that can distinguish between fat and water protons thank to their different magnetic resonance properties. Since fat tissues have higher carbon/hydrogen content than other soft tissues and bones, they can be easily assessed by Computed Tomography (CT) as well. Interestingly, MRI also discriminates between white and brown adipose tissue (BAT); the latter has long been regarded as a potential target for anti-obesity drugs because of its ability to enhance energy consumption through increased thermogenesis. Positron Emission Tomography (PET) performed with ^18^F-FDG as glucose analog radiotracer reflects well the metabolic rate in body tissues and consequently is the technique of choice for studies of BAT metabolism. This review will focus on the main, non-invasive imaging techniques (MRI, CT, and PET) that are fundamental for the assessment, quantification and functional characterization of fat deposits in small laboratory animals. The contribution of optical techniques, which are currently regarded with increasing interest, will be also briefly described. For each technique the physical principles of signal detection will be overviewed and some relevant studies will be summarized. Far from being exhaustive, this review has the purpose to highlight some strategies that can be adopted for the *in vivo* identification, quantification, and functional characterization of adipose tissues mainly from the point of view of biophysics and physiology.

## Introduction

### Fat Tissue: General Characteristics

Fat tissue, or adipose tissue, is a type of connective tissue whose predominant cell type is the fat cell, or the adipocyte. In mammals there are two types of fat tissues: the white adipose tissue (WAT) and the brown adipose tissue (BAT) (Cinti, 2000). BAT is characterized by special fat cells showing a multilocular lipid deposit, wherein thermogenesis takes place by “burning” fatty acids in uncoupled mitochondria (Cannon and Nedergaard, 2004). Instead, WAT cells typically present one lipid vacuole and are the major site for body lipids storage, especially triglycerides, serving as an energy deposit from which fatty acids may be delivered through the bloodstream to meet the body’s energy requirements during fast or intense, enduring physical activity. Moreover, fat cells are plastic enough so that fat tissue may serve as an aid to prevent trauma injuries; fat cells may act as a heat insulator layer as well. Other cell types are found in fat tissue, e.g., preadipocytes, endothelial cells, pericytes (Rosen and Spiegelman, 2000), immune cells (lymphocytes, T-cells, macrophages, neutrophils), and multipotent stem cells (Tchoukalova et al., 2004; Carmon et al., 2008; Kintscher et al., 2008; Cawthorn et al., 2012). Overall, these cell types over number fat cells in adipose tissue; nevertheless, fat cells represent about 90% of tissue volume due to their large size ranging 20–200 microns. Fat tissue also represents an endocrine tissue by producing a variety of adipokines (e.g., leptin, visfatin, adipolin, and many others). Adipokines act at the autocrine/paracrine and endocrine level (Ronti et al., 2006). Adipokines participate in the regulation of glucose and lipid metabolism, energy homeostasis, feeding behavior, insulin sensitivity, and adipogenesis; they are also involved in the regulation of vascular function and coagulation (Romacho et al., 2014). Fat tissue also has a role in the immune function. In non-obese subjects, immune cells resident in fat tissue have housekeeping functions such as apoptotic cell clearance, extracellular matrix remodeling, and angiogenesis (Mathis, 2013). In obese subjects, excess of adipocytes produces danger signals mimicking bacterial infection, and drives a prototypic T helper 1 inflammatory response (Grant and Dixit, 2015).

### Fat Tissue: Body Distribution

Fat tissue is distributed in several discrete anatomical deposits in the body (Shen et al., 2003). It is well known that women generally show higher adiposity than men. Moreover, there is a sexual dimorphism in fat tissue distribution, men accumulate more fat in the trunk region and women accumulate more in the gluteofemoral region (Geer and Shen, 2009). Ethnicity is also a factor that influences body fat distribution (Carroll et al., 2008). Over the last decades it became clear that adipose tissue is not a single homogeneous compartment; instead, it is a tissue with specific regional depots whose biological functions may vary (Vanderburgh, 1992; Schoen et al., 1996) and individual adipose tissue deposits are more closely correlated with physiological and pathological processes than total adipose tissue mass (Kelley et al., 2000). In humans, the largest fat tissue deposit is the subcutaneous adipose tissue, representing about 80% of body fat (Frayn and Karpe, 2014). Within subcutaneous adipose tissue different sub-deposits have been identified: the abdominal, gluteal, and femoral sub-deposits. The subcutaneous adipose tissue is divided into superficial and deep layers by the *fascia superficialis*. These layers have different functions and different correlations with metabolic complications of obesity (Smith et al., 2001). Visceral adipose tissue is found around organs in the thoracic and, to a greater extent, in the abdominal cavity. It has morphological and functional differences from subcutaneous adipose tissue. For example, visceral adipose tissue contains larger, insulin-resistant adipocytes, presents a well-developed vasculature and abundant innervation, and it is more sensitive to lipolysis. On the other hand, subcutaneous fat shows smaller, insulin-sensitive fat cells with less developed vasculature and nerve supply (Mårin et al., 1992; Bjorntorp, 2000). Subcutaneous and visceral adipose tissues have peculiar adipokine expression and secretion profiles (Kershaw and Flier, 2004). Visceral adipose tissue releases larger amounts of pro-inflammatory cytokines in the vena porta, which directly impacts liver metabolism; in its turn, subcutaneous adipose tissue releases more leptin and larger amounts of adiponectin, an anti-inflammatory and insulin-sensitizing adipokine (Ibrahim, 2010). Therefore, the ability to identify and quantify different adipose deposits non-invasively, which is a peculiar feature of *in vivo* imaging techniques, appears relevant to obesity research.

Triglycerides may also be deposited within cells of non-adipose tissue that normally contain only small amounts of fat; this is called ectopic fat deposition (Shulman, 2014) and it is usually associated with obesity and positive energy balance. Excess lipids can accumulate in the heart, liver, skeletal muscle, and pancreas probably because the adipose tissue is no longer capable of sequestering nutritional lipids. Ectopic fat deposits have been claimed as risk factors for disease development (Mathieu et al., 2014).

### Fat Tissue: Body Composition Analysis

In the context of body composition analysis, fat (adipose) tissue and body fat are not synonymous. When the five levels (I, atomic; II, molecular, III, cellular; IV, tissue/system; V, whole body) approach to body composition is adopted (Wang et al., 1992), fat tissue is considered at level IV as a specialized loose connective tissue containing lipid-laden adipocytes as well as intra- and extracellular water, interstitial cells, blood vessels, etc. Instead, body fat at level II is considered as the total mass of body lipids (especially triglycerides). These lipids are mainly contained in the lipidic droplets of fat tissue adipocytes, but also in intermuscular adipocytes, in sparse adipocytes of interstitial tissue as well as in muscle cells, hepatocytes, cell membranes, etc. As a result, the total amount of fat tissue and body lipids may be similar, but not identical (Tothill et al., 1996). Traditionally, body fat has been evaluated with a two-compartment model, i.e., subdividing body mass in fat mass and fat-free mass (lean mass). For decades, the standard method for determining fat mass has been underwater weighing; later on, body water dilution, bioelectrical impedance, and air-displacement plethysmography have been used (Pietrobelli et al., 1998) until three-dimensional anthropometry (Wells et al., 2008; Giachetti et al., 2015). In parallel, several imaging techniques emerged as useful tools for body composition analysis on a multi-compartmental basis, e.g., magnetic resonance imaging (MRI), computed tomography (CT), ultrasounds (US), and positron emission tomography (PET).

### Adipose Tissue as a Target for Anti-obesity Drugs

Obesity is nowadays recognized as a real pathological state requiring pharmacological and in some cases even surgical treatment. Several drugs for obesity treatment have been marketed over the past few years, but their efficacy was limited and severe adverse effects have often been reported. Anti-obesity drugs on the market or in clinical trials have been recently reviewed (Giordano et al., 2016). Their mechanism of action broadly falls in two categories: drugs that reduce nutrients absorption and anorexic drugs that act on the central nervous system to induce sense of satiety. An innovative mechanism of action for anti-obesity drugs has been more recently proposed and it consists in promoting conversion of WAT into BAT (Giordano et al., 2016). Indeed, it is well known that adult WAT can convert into BAT (browning), this conversion being triggered by several conditions including low ambient temperature, physical exercise or β3-adrenoceptor stimulation. White adipocytes converted to brown adipocytes are considered a new type of brown fat cells named beige adipocytes. A number of molecular targets that can potentially promote conversion of WAT into BAT have been identified recently and reviewed (Giordano et al., 2016). Interestingly, the target of such adipose conversion is the visceral WAT, which is inflamed in obese subjects and possibly involved in most of the adverse clinical correlates of obesity, e.g., metabolic syndrome. In the context of pharmacological research in anti-obesity drugs, suitable animal models and related imaging techniques are needed. In this review, we will examine the contribution of different imaging techniques to the issue of *in vivo* fat tissue identification, quantification, and functional characterization. While most of such imaging studies have been carried out in humans, longitudinal, mechanistic investigations of the metabolic and health correlates of excess/paucity of body fat are better performed in experimental animals. Small rodents, especially mice and rats, are extensively used in preclinical studies aimed at clarifying the causes and mechanisms of metabolic syndrome, diabetes, and obesity (Rees and Alcolado, 2005; Varga et al., 2010; Kanasaki and Koya, 2011) as well as in studies aimed at defining the effect of candidate drugs. Since BAT appears a potential new target tissue for anti-obesity drugs, special attention will be devoted to imaging techniques allowing for identification, quantification, and functional state assessment of BAT.

## Imaging Techniques for Detection of Fat Tissues in Preclinical Studies

Preclinical imaging has been widely employed in the study of obesity and metabolism. Preclinical protocols for assessing whole body and regional adipose tissue content have been reported using MRI, CT, and Dual-energy X-ray Absorptiometry (DXA) (Sjögren et al., 2001; Sasser et al., 2012; Metzinger et al., 2014). While DXA has been used to accurately estimate lean and fat mass in rodents, as a bi-dimensional radiological technique, it does not provide tridimensional information on fat volume and distribution (Luu et al., 2009); accordingly results obtained with DXA are not reported in this review. The interested reader is referred to the excellent reviews from Albanese et al. (2003) and Toombs et al. (2012). US has been used as an effective and economic technique to assess body composition in humans although its accuracy is strongly dependent on the operator proficiency (Wang et al., 2014). Moreover, applications of US to animal models of obesity and metabolic disorders are still limited; consequently US will not be discussed in this review. ^18^FDG-PET has been employed in studies of BAT and metabolic disease (van der Veen et al., 2012). Preclinical imaging provides longitudinal imaging of obesity models, studies of molecular mechanisms of obesity and evaluation of candidate obesity therapeutics. Far from being exhaustive, this review has the purpose to highlight some strategies that can be adopted for the *in vivo* identification, quantification, and functional characterization of adipose tissues mainly from the point of view of biophysics and physiology.

### MR-Techniques

**FIGURE 1:**
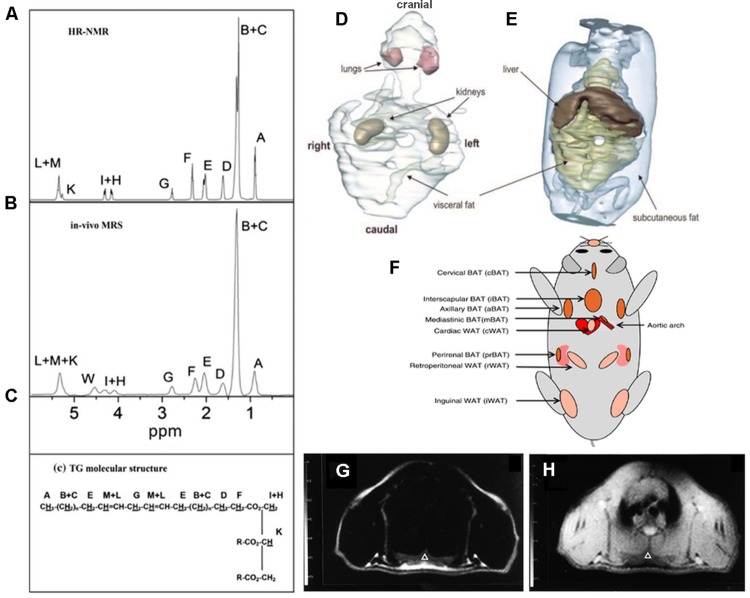
**Magnetic Resonance Spectroscopy and Imaging of fat tissues.** Representative *ex vivo* **(A)** and *in vivo* **(B)** spectra of inguinal white adipose tissue. The areas under each peak are proportional to the number of protons in a given chemical environment within a triglyceride molecule **(C)** (from Giarola et al., 2011 reprinted with permission). **(D,E)** 3D reconstruction of MR images of whole trunk and selected structures in an ob/ob mouse showing the space distribution of fat deposits (from Calderan et al., 2006, reprinted with permission); **(F)** localization of the principal BAT depots and some WAT depots in the mouse (from Vosselman et al., 2013, reprinted with permission). Chemical shift selective transversal MR images at the level of the interscapular BAT in a living rat: **(G)** fat image; **(H)** water image. IBAT is marked by a triangle (from Lunati et al., 1999, reprinted with permission).

Magnetic Resonance Imaging and Magnetic Resonance Spectroscopy (MRS) are currently considered to be the most comprehensive tools for quantification of fat in living organisms (Hu and Kan, 2013). Of note, MRI and MRS can be performed by a single instrument and within the same experimental setup. MRI and MRS have the same underlying concepts and exploit the difference in the resonance frequency between water and fat protons, a phenomenon known as chemical shift. Representative *ex vivo* and *in vivo* spectra obtained from WAT in mice are reported in **Figures 1A,B** where several proton resonances attributable to different chemical environments can be distinguished. The water proton resonance occurring at 4.7 ppm is barely visible in the WAT tissue. The signal at 1.3 ppm corresponds to the CH_2_ methylene protons of the lipid chain, shown in **Figure 1C**, and represents the bigger component in the triglyceride spectrum (Bley et al., 2010). The spectrum reported in **Figure 1B** has been obtained after carefully shimming over a small Volume-of-Interest (VOI) and its spectral resolution is comparable to the resolution of the *ex vivo* spectrum reported in **Figure 1A**. However in most experimental setups only a few of the triglyceride spectral lines can be detected (see e.g., the typical liver spectrum in Figure 1 of Reeder et al., 2011). Consequently, in most of applications of MRS for determination of water/fat content, two peaks are considered: the water peak (4.7 ppm) and the CH_2_ protons of the lipid chain peak (1.3 ppm), the latter containing more than 10 times the signal of any other fat peak (Bley et al., 2010). The fat and water content of a given tissue can be estimated from proton spectra as the ratio between the area of the water and CH_2_ protons peaks.

Proton spectra acquired with high spectral resolution are used to chemically characterize lipid molecules *in vivo* (Giarola et al., 2011; Mosconi et al., 2011; Ye et al., 2012) in terms of unsaturation, poly-unsaturation index, and mean chain length. Quantification of protons corresponding to a certain chemical species requires suitable spectra analysis generally performed using commercial software (Giarola et al., 2011; Mosconi et al., 2011, 2014). To the best of our knowledge, ours was one of the first groups to suggest that *in vivo* MRS can provide information about fat composition in living animals (Lunati et al., 2001). Afterward, Mosconi et al. (2011) demonstrated that WAT in obese Zucker rats, a widely used experimental model for diabetes and obesity, is characterized by lower unsaturation and polyunsaturation index compared with controls (lean Zucker rats) thereby confirming the hypothesis that obese and lean Zucker rats have different adipose tissue composition. Ye et al. (2012) applied MRS to study lipids in the liver of ob/ob mice at 9.4 T and demonstrated that the mean chain length was significantly longer and the fraction of monounsaturated lipids higher in ob/ob mice than in control.

The fact that water and methylene lipid spectral lines are separated by about 3.5 ppm (corresponding to 420, 700, 1043 Hz at 3, 4.7, and 7 T, respectively) is the base for fat/water selective imaging. Several reviews have been recently published describing details of MRI techniques used to obtain water and fat separation (Bley et al., 2010; Reeder et al., 2011; Hu and Kan, 2013). Briefly, these techniques can be divided into frequency-selective or phase-selective. In frequency-selective imaging, the excitation pulse is shaped to selectively excite water or fat proton signal prior to acquisition. In this case the signal will be detected only from water or fat. In a different approach, frequency-selective pulses are first applied to suppress water or fat signal and then the signal of the unsuppressed chemical species is acquired. Fat selective excitation has been used to characterize the water/fat content of interscapular BAT and visceral WAT in the laboratory animal (Lunati et al., 1999) and it is routinely applied to suppress fat signal in clinical examinations where the high intensity signal coming from fat can mask the signal coming from other organs or pathologies.

The same physical property, i.e., chemical shift of water and methylene protons, is exploited in the phase-selective imaging methods originally proposed by Dixon (1984). A review of methods and applications of phase selective imaging has been recently published (Hu and Kan, 2013).

Compared to tissue water protons, fat protons are characterized by short longitudinal relaxation time (T1) and long transversal relaxation time (T2). Indeed, the T1 relaxation time of fat tissues is one of the shortest measurable *in vivo* (Hu and Kan, 2013). Accordingly, fat tissue appears brightest in T1 weighed images thereby allowing for easy quantification of fat volume trough threshold-based segmentation of images (Hu and Kan, 2013). This is defined the relaxometry-based approach while the above mentioned methods, based on the chemical shift difference between water and fat proton, are defined chemical shift-based approaches. Relaxometry-based approaches are widely applied in small animal imaging. Calderan et al. (2006) used this approach to study the fat distribution in ob/ob mice trough segmentation of T1 weighted images and 3D reconstruction of fat depots and internal organs (**Figures 1D,E**). The same approach was applied in longitudinal studies of body fat accumulation in cathepsin K null mice and its wild type during treatment with high fat diet for 12 weeks (Funicello et al., 2007). The above mentioned differences in T1 and T2 values between water and fat protons permit analysis of body composition in awake small laboratory animals with simple acquisitions of the nuclear magnetic resonance (NMR) signal in the time domain from the whole body. Small bench top NMR instruments are now available that allow precise measurement of lean, fat, and free fluid content in laboratory animals which are widely used in pharmaceutical as well as in diabetes and obesity research. Such approach was used to measure body mass composition (fat, lean, and free fluids) in Ghrelin-receptor null transgenic mice in comparison to wild type, in order to comprehend the action mechanism of a new drug acting as a selective and potent antagonist of the Ghrelin receptor (Costantini et al., 2011).

The anatomical localization of the principal BAT and some WAT deposits are shown in **Figure 1F**. It is noteworthy that MRI can distinguish between BAT and WAT. It has long been known that MRI findings correlate with ultrastructural patterns of BAT in laboratory animals (Osculati et al., 1991); this study was performed at different ages indicating that MRI is a reliable tool to investigate BAT tissue along its age-associated changes. Moreover, MRI allows for accurate determination of BAT deposits volume in living animals and reveals tissue changes associated with temperature manipulation, findings confirmed by histological and ultrastructural analysis (Sbarbati et al., 1991). Modification induced in BAT by interferon were studied with MRI and confirmed by histology and TEM. In particular, in treated mice, the interscapular BAT (iBAT) was found to be slightly enlarged and showed inhomogeneous areas of lipid accumulation (Sbarbati et al., 1995). As shown in **Figures 1G,H** unequivocal definition of BAT deposits can be obtained using chemical shift selective imaging for fat and water protons; the experimental paradigm was validated in rats at 4.7 T (Sbarbati et al., 1997). MRI with fat selective excitation and correction for the T2 relaxation time was applied to quantify fat and water content in the iBAT of rats at 4.7 T in order to obtain lipidic maps of this tissue (Lunati et al., 1999). Chen et al. (2012) demonstrated the feasibility of estimating BAT volume and metabolic function *in vivo* in rats at a 9.4 T using sequences available from clinical MR scanners. Specifically, they measured the volume distribution of BAT with MRI sequences showing strong fat-water contrast, and investigated BAT volume by utilizing spin-echo MRI sequences. MRI-estimated BAT volumes were compared with the mass of the excised samples. Moreover they were able to map the hemodynamic responses to changes in BAT metabolism induced pharmacologically by the β3-adrenergic receptor agonist, CL-316,243 in comparison with PET ^18^F-FDG imaging, demonstrating the feasibility of BAT volume and functionality assessment with routinely used MRI sequences. It should be mentioned that over the past several years, BAT activity *in vivo* has been primarily assessed by PET (or PET-CT) scan following ^18^F-FDG administration to measure glucose metabolism (see “PET imaging”). Another approach, the two-point magnitude MRI, based on a slightly modified standard MRI protocol, has been proposed to visualize mouse BAT differentiating it from surrounding WAT at 11.7 T (Lindenberg et al., 2014).

In order to discriminate between BAT and WAT in lean and ob/ob mice, T2* relaxation times and proton density fat-fraction values were measured in the two tissues (Hu et al., 2012). To determine differences in fat-signal fraction (FF) from chemical-shift-encoded water-fat MRI of iBAT, Smith et al. studied mice housed at different ambient temperatures. They found that lowering temperature leads to a significantly reduction in BAT-FF, in accordance with the expected BAT involvement in thermogenesis (Smith et al., 2013). Comparison between BAT and WAT fat fractions in ob, seipin, and Fsp27 gene knockout mice by chemical shift-selective imaging and (1)H-MR spectroscopy was reported by Peng et al. (2013). Finally, the effect of different diets on composition of intra-hepatocellular lipids as well intra-abdominal, subcutaneous and total adipose tissue, and BAT was measured *in vivo* with whole body 3D imaging (Bidar et al., 2012).

Several novel approaches have recently been explored to validate the *in vivo* quantification of BAT with MRI. In particular a dual-echo sequence, both with and without spectral presaturation inversion recovery (SPIR) fat suppression, was used at 1.5 T and validated through comparison with histology and ^18^FDG-PET (Holstila et al., 2013). A new MRI method combined intermolecular double-quantum coherence and the chemical shift-encoded Dixon method in order to enable detection of BAT cells mixed to other cells. The contrast in this technique depends on the water-to-fat ratio at the cellular size scale, which is smaller than the imaging voxel size (Bao et al., 2013). Another method, based on the normally invisible intermolecular multiple-quantum coherence (1)H MR signal has been proposed (Branca et al., 2013). This method does not require special hardware modifications and can overcome the partial volume effect. Moreover, it exploits the characteristic structure of BAT adipocytes to selectively image them, even when they are intimately mixed with other cell types. The method was validated in mice using PET scans and histology. The patterns of oxygen consumption/perfusion were imaged by using blood-oxygen-level-dependent MRI upon BAT activation (Khanna and Branca, 2012) and a well-localized signal drop in BAT found, related to a substantial increase in oxygen consumption and the consequent increase in blood deoxyhemoglobin levels. Sbarbati et al. (2006) used contrast enhanced MRI to study the effect of adrenergic stimulation on iBAT: adrenergic stimulation performed 40 s before MRI lead to a significantly higher enhancement of signal intensity in iBAT compared to unstimulated tissue, indicating that BAT stimulation is accompanied by increased blood flow.

The use of hyperpolarized nuclei for the identification and assessment of BAT function was proposed by two different groups. The first one (Lau et al., 2014) investigated the feasibility of using hyperpolarized (13)C imaging to quickly (<1 min) identify activated BAT in an *in vivo* rodent model following an infusion of pre-polarized [1-(13)C] pyruvate. Using hyperpolarized xenon gas, the second one (Branca et al., 2014) demonstrated a greater than 15-fold increase in xenon uptake by BAT during stimulation of thermogenesis, thereby, obtaining background-free maps of the tissue in both lean and obese mouse phenotypes.

### Computed Tomography

X-ray computed tomography (also called X-ray CT or simply CT) is based on the combination of many X-ray images taken from different angles to produce tomographic images of a scanned object. For *in vivo* acquisitions, the most used construction principle involves scanners with X-ray detector and the radiation source mounted on a gantry that is rotated around the examined object. The majority of the marketed scanners use nano-microfocus X-ray tubes in which an electron beam is focused by several magnetic lenses onto a focal spot of 1–10 μm which interacts with a transmission target to produce the X-ray radiation. Passing through the samples, X-rays are differently absorbed by different materials; the transmitted radiation reaches scintillator crystals and is converted in light signals. The light is then lead by tapered glass fibers (Schambach et al., 2010) to a detector which is often a charge coupled device camera (CCD) and transformed in a digital image. A large series of two-dimensional radiographic images are taken around a single axis of rotation and trough suitable algorithms a three-dimensional image of the inside of the object can be generated. MicroCT systems are capable of volumetric CT analysis with isotropic voxels spacing 50–100 μm (Holdsworth and Thornton, 2002).

**FIGURE 2:**
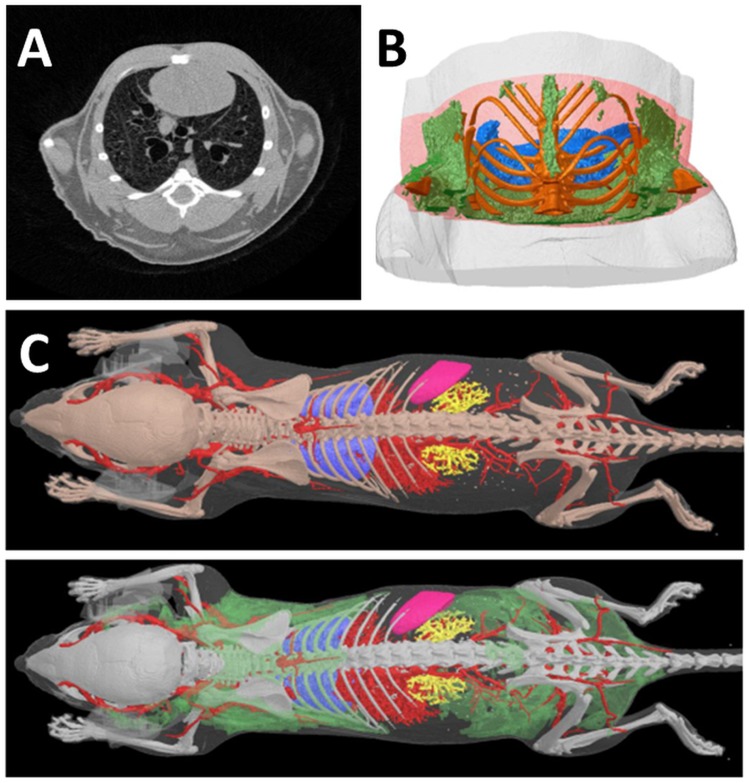
**Computed Tomography of fat tissues. (A)** In a reconstructed cross section of a microCT scan of a mouse thorax, the four tissues which are most readily distinguished in microCT images on the basis of differing x-ray attenuation are bone, lung, fat and “lean” or non-fat soft tissue. All four tissues are clearly visible in this image; the adipose tissue appears as the darker gray regions near the periphery of the thorax. Note that in this image lighter color means higher x-ray attenuation and “density,” while darker color means low x-ray attenuation. Bone is saturated to white by narrowing of the reconstruction contrast limits to enhance soft tissue visual contrast. **(B)** A segment VOI of the mouse thorax selected for fat analysis relative to a lung landmark (branching of trachea) shows adipose tissue in green, lungs blue, bone gold. **(C)** Adipose tissue (green) in the lower surface rendered model image can be segmented and visualized on the basis of its lower X-ray density vs. “lean” soft tissues. (Courtesy of Bruker microCT NV, Kontich Belgium).

The different ability of the anatomical structures to attenuate the X-ray beam is responsible of the contrast in CT images. The adipose tissue has different X-ray attenuation compared to other soft tissues, and thus it has a distinct density in microCT images (**Figure 2A**). Using microCT scanners with X-ray photon energies in the range 15–75 keV the principal modes of X-ray attenuation are photoelectric absorption and Compton scattering. Which mode predominates depends on the photon energy and the atomic number (Z) of the absorbing element (Evans, 1955, chapter 25, Figure 1.1). Photoelectric absorption predominates at low photon energies and in heavier elements, while higher photon energies and lower Z nuclei favor Compton scattering. Photoelectric absorption in materials is proportional to Z^4^, this means an acute sensitivity of photoelectric absorption to elemental composition. Instead Compton scattering has unitary proportionality to Z. This difference is important: it implies that photoelectric absorption gives much stronger absorption image contrast, based on material element composition, than Compton scattering. The elemental composition of soft tissue is predominantly hydrogen (H), carbon (C), nitrogen (N), and oxygen (O).

Elemental ratios in some representative biological tissues are shown in **Table 1**. The content of C and O in fat is 57 and 30%, respectively, while in muscle (typical of soft tissues other than fat) the content of C and O is 12 and 73%, respectively.

**Table 1:** Biological tissue elemental ratios by mass percentage (modified from Johns and Cunningham, 1983).

	H	C	N	O	Na	Mg	P	S	K	Ca
Atomic number Z	1	6	7	8	11	12	15	16	19	20
Fat	11.2	57.3	1.1	30.3				0.006		
Water	11.2			88.8						
Muscle	10.2	12.3	3.5	72.9	0.08	0.02	0.2	0.5	0.3	0.007
Bone	6.4	27.8	2.7	41.0		0.2	7.0	0.2		14.7

The large difference in the carbon to oxygen ratio between fat and lean tissues accounts for the difference in X-ray absorption at X-ray energies where photoelectric absorption is the predominant interaction mechanism, i.e., at photon energies less than about 30 keV. C, N, and O are close to the boundary between predominance of photoelectric absorption and Compton scattering, at photon energies less than 50 keV so they can attenuate X-rays by both modalities. Decreasing X-ray photon energy, photoelectric absorption is favored and the Z-based contrast will increase. This means that fat can be imaged by microCT by using an appropriate X-ray energy without causing excessive radiation dose to the animal.

Computed tomography images can be treated with a set of software procedures (thresholds detection, segmentation) in order to enhance contrast, i.e., increase difference among anatomical structures enabling subsequent computational analysis. After segmentation of 2D images, volumetric reconstructions of different organs, including fat, can be obtained as reported in **Figures 2B,C**.

Computed tomography imaging has natural applications to bone imaging, with a special focus on bone architectures, bone density and vascular imaging, thanks to the implementation of novel vascular contrast agents (Holdsworth and Thornton, 2002). Moreover, it is also able to provide three-dimensional density maps with sufficient contrast to distinguish adipose tissue from other tissues, fluids and cavities without contrast agents (Luu et al., 2009). Noteworthy, it can not only measure the total volume of adipose tissue within an animal, but can also identify and quantify very small volumes of fat residing in discrete deposits (Luu et al., 2009). Indeed, acquiring high-resolution images based on the physical densities of the object allows discrimination of subcutaneous adipose tissue and visceral adipose tissue (Judex et al., 2010). As a non-invasive, *in vivo* technique CT provides measurement of the total, visceral, and subcutaneous adiposity in longitudinal studies (Luu et al., 2009). Adipose volumes determined by microCT and the weight of the explanted fat pads are highly correlated, demonstrating that CT can accurately monitor site-specific changes in adiposity (Judex et al., 2010). From the experimenter’s point of view, it is important to note that voxel densities of fat are relatively uniform throughout the adipose tissue and partial volume effect in adipose tissue is less important than in bony structures. Instead, a greater effort is needed to set accurately voltage and current of the X-ray source to optimize the contrast.

Although fat tissue is spanned over the entire body, it was demonstrated that differences in total fat volume across various animal species are congruent with differences in their abdominal fat mass (Rubin et al., 2007). So scanning the entire mouse may not be necessary and only the abdominal region can be acquired saving acquisition time and dose administered to the patient (Judex et al., 2010). For a quantitative analysis of discrete fat deposits, manual drawing of contour lines is very time consuming and does not yield adequate precision and accuracy; algorithms have been written and are now available based on automatic edge detection in the images, in order to obtain reproducible results (Judex et al., 2010). MicroCT can also allow determining the degree of fat infiltration in liver by measuring the liver-to-spleen density ratio in a specific region around the intervertebral disk between the 13th thoracic and first lumbar vertebrae (Judex et al., 2010).

MicroCT was used to investigate the effect of high frequency and low intensity mechanical signals on adipogenesis in mice (Rubin et al., 2007). This study demonstrated that 15 weeks of brief, daily exposure to high-frequency mechanical signals of a magnitude well below that which would arise during walking, inhibits adipogenesis by 27% in C57BL/6J mice.

Using microCT scanning, a reduced percentage of adipose mass associated with decreased adipocyte cell size was found in mice null for Fyn (a member of the Src family of non-receptor tyrosine kinases). Such reduction was accompanied by a substantial reduction in fasting plasma glucose, insulin, triglycerides and free fatty acids, concomitant with decreased intrahepatocellular and intramyocellular lipid accumulation. For the quantification of total fat volume inside a volume of interest, freshly harvested fat pad from similar mouse, with identical scan settings, were imaged in order to identify the upper and lower thresholds useful to separate adipose tissues from other tissues and fluids (Bastie et al., 2007).

A study on BAT was conducted on rats exposed to room temperature (23–24°C) as the control condition and after 4 h of cold exposure (4°C), which is known to activate BAT. The CT Hounsfield units (which are related to the tissutal radiodensity) of BAT resulted higher (whiter density in the images) under the activated condition than under the control condition (Baba et al., 2010).

### PET Imaging

Positron emission tomography is a nuclear medicine imaging technique used to reveal functional processes in living organisms. The main applications are in oncology, neuroimaging, cardiology, infectious disease, musculoskeletal imaging, and pharmacokinetics studies. PET is routinely used on humans and, in preclinical applications, on experimental animals. As far as fat imaging is concerned, applications of PET are limited to BAT.

Positron emission tomography instruments are able to detect pairs of gamma rays emitted in living bodies by i.v. injected radiotracers. The radiotracers used in PET contain positron-emitting isotopes, i.e., isotopes decaying by emitting positive charged electrons, called positrons. The positron can travel for about 1 mm in biological tissues before it loses most of its energy and encounters an electron. The interaction annihilates both particles producing two gamma rays emitted in opposite directions. The gamma rays are detected by scintillator crystals (of which the internal ring of the tomograph is made) generating bursts of light revealed by photomultiplier tubes (or avalanche photodiode) coupled with the crystals. Each light burst is then converted in electric signal. The detection of two different, almost simultaneous, events in crystals located approximately at 180 degrees with respect to the center of the tomograph is referred to as a coincidence and the signal is considered due to a real nuclear decay event. Noise or spurious events are attributable to the interaction of cosmic rays or natural radioactivity within the instrument.

The decay time of radioisotopes for PET applications is preferably chosen short enough to reduce the radiation exposure of patients, but at the same time, long enough to allow chemical synthesis and transport from the production site to the imaging facility. The most commonly used radiotracer in PET imaging is 2-deoxy-2-^18^F-fluoro-D-glucose (^18^F-FDG), an analog of glucose, which is labeled with 18 Fluorine. The half-life time of ^18^F is 110 min. ^18^F-FDG is internalized by cells in proportion to their metabolic activity so it accumulates preferentially in cancer cells, brain, heart, and kidney. Inside cells, ^18^F-FDG cannot be further metabolized and it cannot move out of the cell before radioactive decay. Consequently, the distribution of ^18^F-FDG reflects well metabolic rate in body tissues and consequently it is the most relevant radiotracer for studies of BAT metabolism.

Fueger et al. (2006) investigated the uptake of ^18^F-FDG in BAT of mice under different experimental conditions. It is well known that BAT metabolism is activated in mice by low temperature to generate heat. Consequently, the uptake of ^18^F-FDG in BAT was found to be higher at room temperature (21°C) than at thermoneutrality (30°C). ^18^F-FDG uptake by BAT was reduced by fasting the animals overnight, in accordance with the well-known role of BAT in postprandial thermogenesis.

^18^F-FDG uptake in BAT also increases in mice exposed to full-thickness thermal injury (30% of total body surface area), cold stress (4°C for 24 h) or cutaneous wounds (5-fold, 15-fold, and 15-fold, respectively), whereas the uptake in adjacent WAT is unchanged (Carter et al., 2011). Using a thermal imaging camera, a linear relationship between ^18^F-FDG uptake and BAT temperature was demonstrated for the first time in *in vivo* studies (Carter et al., 2011).

^18^F-FDG-PET imaging was used to investigate the diurnal rhythm of glucose uptake in C57Bl/6 mice: glucose uptake in iBAT peaks at approximately 9 h into the light phase of the 12 h light period. This result makes iBAT a candidate site of interaction between metabolic and circadian systems (van der Veen et al., 2012).

**FIGURE 3:**
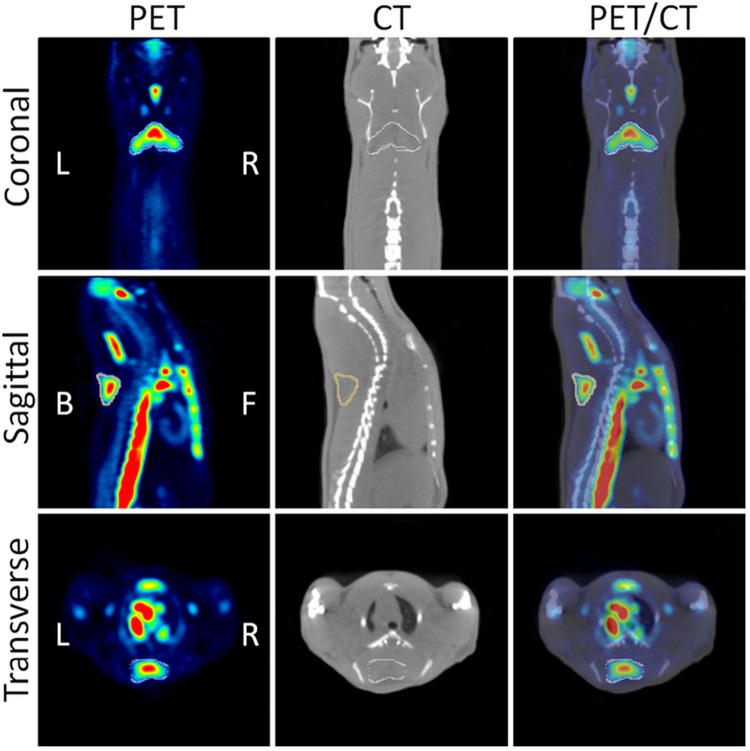
**Coronal, sagittal and transverse views of PET/CT images.** The images show CL316243 activated BATs in Sprague-Dawley rats: PET **(Left)**, CT **(Middle)**, and fused PET/CT **(Right)** (from Mirbolooki et al., 2011, reprinted with permission).

Pharmacological approaches were used to demonstrate that adrenergic pathway activation enhances BAT metabolism in rodents (Mirbolooki et al., 2011, 2013, 2014). BAT is innervated by sympathetic noradrenergic nerve fibers whose stimulation activates β3-adrenoreceptors in the target tissue resulting in enhancement of glycolysis. This probably increases the synthesis of cyclic AMP and the overexpression of uncoupling protein-1 (UCP1). In the first study, BAT activation was induced by administration of CL316243, a β3 adrenoceptor agonist in rats, and evaluated by ^18^F-FDG-PET imaging (Mirbolooki et al., 2011). CL316243-induced activation of BAT was clearly visible in PET images, in particular in the interscapular, cervical, periaortic, and intercostal BAT deposits. The uptake of ^18^F-FDG was enhanced by 12-fold in comparison to control animals, while low temperature (8°C for 120 min) increased the ^18^F-FDG uptake only 1.1-fold (**Figure 3**). The uptake of ^18^F-FDG in activated iBAT was greatly reduced (96.0%) by administration of the β-blocker propranolol. These results were confirmed by *ex vivo* ^18^F-FDG autoradiography and histology. In a second study, the effect of presynaptic activation with atomoxetine on BAT metabolism was evaluated in rats (Mirbolooki et al., 2013). The existence of norepinephrine transporters in BAT was previously suggested by *in vivo* and *ex vivo* evaluations using ^11^C-MRB, a highly selective norepinephrine transporter ligand for BAT imaging at room temperature in rats (Lin et al., 2012). It is noticeable that for this study the positron emitter ^11^C with 20.3 min half-life was used. The results obtained in the studies previously mentioned were confirmed and extended to mice using three pharmacological approaches (atomoxetine, CL316243, and forskolin, an adenylyl cyclase enzyme activator) in a third study (Mirbolooki et al., 2014). Atomoxetine increased ^18^F-FDG uptake of iBAT 1.7-fold vs. control mice. CL316243 increased ^18^F-FDG uptake fivefold in IBAT, 2.4-fold in WAT and 2.7-fold in muscle vs. control mice. Finally, forskolin increased the uptake 1.9 in IBAT, 2.2-fold in WAT and 5.4-fold in heart compared to controls.

Beyond its role in thermal homeostasis (during both acute stress and cold acclimation), BAT is probably involved in energy homeostasis as a site of postprandial thermogenesis. The ablation of the essential protein for heat production in BAT namely, the uncoupling protein-1 (UCP-1), leads to an obese phenotype in mice housed at thermoneutral temperature (Feldmann et al., 2009). BAT was found to be involved in plasma triglyceride clearance (Bartelt et al., 2011) and glucose homeostasis (Guerra et al., 2001; Gunawardana and Piston, 2012). Accordingly to Vosselman et al. (2013) these results highlight the antiobesity role of BAT in rodents, as well as its potential in obesity-related metabolic diseases (diabetes and cardiovascular disease).

Activation of BAT can be a new strategy to combat obesity and diabetes mellitus (DM). Wu et al. (2014) obtained models of obesity by feeding mice with a high fat diet for 8 weeks and models of DM by administration of streptozotocin to obese mice. Both obese and DM mice showed lower ^18^F-FDG uptake in iBAT compared to controls. After 2 weeks of treatment with BRL37344 (a β3-adrenergic receptor agonist) the uptake was significantly increased in both animal models with a decrease of blood glucose levels and substantial weight loss in obese mice. Levothyroxine (the synthetic thyroid hormone) increased ^18^F-FDG uptake in both obese and control mice, but not in DM mice. These results demonstrate that inhibition of BAT function found in obese and DM mice can be reversed by β3-adrenergic receptor agonist or thyroid hormone administration (Wu et al., 2014) and BAT activation may effectively lead to weight loss and blood glucose lowering.

To evaluate the significance of β3-adrenoreceptor agonist-induced BAT activation in obesity, a useful rat model is represented by the Zucker lean and obese rats. The effect of CL316243 administration on the BAT ^18^F-FDG uptake was investigated in both genotypes, resulting in fourfold increase of glucose uptake in Zucker lean with respect to saline-administered control rats and only a twofold increase in Zucker obese rats. The reduced CL316243 activation is consistent with the lower β3-adrenoreceptor levels in Zucker obese with respect Zucker lean rats (Schade et al., 2015).

A norepinephrine analog labeled with the 11C isotope ((11)C-meta-hydroxyephedrine, 11)C-MHED) was used to investigate the sympathetic nervous system (SNS) activity in BAT of lean and dietary obese mice, demonstrating that 11C-MHED is a specific marker of the SNS-mediated thermogenesis in BAT deposits, and that this radiotracer can detect *in vivo* the WAT-to-BAT conversion (Quarta et al., 2013).

Recently, concomitant application of ^11^C-acetate, ^18^FDG, and ^18^F-fluoro-thiaheptadecanoic acid (^18^FTHA) has been reported in order to characterize BAT alterations in both clinical (Ouellet et al., 2012) and preclinical studies (Labbé et al., 2015, 2016). In preclinical studies, the effects of cold on BAT were investigated using ^18^F-FDG (for glucose uptake), ^18^FTHA (for non-esterified fatty acid–NEFA- uptake), and 11C-acetate (for oxidative activity). The experiment was performed in rats, adapted to 27°C, which were acutely subjected to cold (10°C) for 2 or 6 h and in rats chronically adapted to 10°C for 21 days, which were returned to 27°C for 2 or 6 h. Cold exposure (acute and chronic) led to increases in BAT oxidative activity, which was accompanied by concomitant increases in glucose and NEFA uptake (Labbé et al., 2015). The same radiotracers were used to investigate the metabolic activity of iBAT and “beige adipose tissue” in mice exposed to cold or to an adrenergic agonist (CL) (Labbé et al., 2016) extending the results found in humans (Ouellet et al., 2012).

Finally, an experimental protocol for BAT functional imaging with ^18^F-FDG in mice was proposed by Wang et al. (2012) in order to standardize the imaging procedures, to uniform the post-images analysis and to quantify the FDG uptake in BAT as percentage of the injected dose per gram of tissue. The described method, which is based on a small animal- micro-PET/CT system, can be applied to screening drugs/compounds that modulate BAT activity, or to identify genes/pathways that are involved in BAT development and regulation in preclinical studies.

### Other Imaging Techniques: Cerenkov Luminescence Imaging and Fluorescence Imaging

It has been recently reported that beta+ or beta- emitting radionuclides can be detected in living animals by Cerenkov Luminescence imaging (CLI) using standard optical imaging instrumentation (Boschi and Spinelli, 2014; Spinelli and Boschi, 2015). This methodology relies on the well-known Cerenkov effect. Briefly, while traveling in the biological tissues, the emitted particles polarize molecules of the medium which emit electromagnetic waves relaxing back to the equilibrium. If the particle travels with a speed greater than the speed of light in the medium, the electromagnetic waves interfere constructively producing a shock front, which can be detected in the UV-visible-near infrared range as Cerenkov radiation.

**FIGURE 4:**
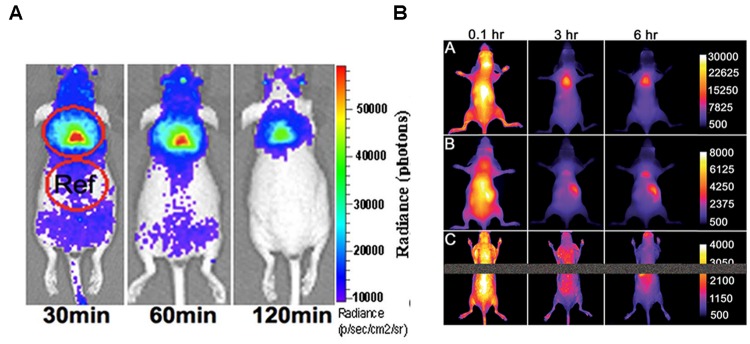
**(A)** Cerenkov luminescence images of a mouse at 30, 60, 120 min after10.3 MBq ^18^F-FDG intravenous injection (from Zhang et al., 2013, reprinted with permission); **(B)** Fluorescence images of living SKH1 hairless mice after intravenous 10 nmol dose of SRFluor680 [A], IR780 [B], or Nile Red [C] and imaged periodically over a period of 6 h. The fluorescence pixel intensity scale bar applies to all images in the same row (arbitrary units) (from Rice et al., 2015, reprinted with permission).

Brown adipose tissue and its activation can be studied by optical techniques, via CLI, as shown by Zhang et al. (2013) after administration of ^18^F-FDG in mice. They demonstrated that CLI is able to detect iBAT *in vivo*. Data were confirmed by *ex vivo* radioactivity measurements; representative images are shown in **Figure 4A**. Using norepinephrine as a stimulator, they found that norepinephrine-treated mice show significantly higher CLI signals compared to untreated mice. Moreover, in treated animals they observed an increase of the signal under short (5 min) isoflurane anesthesia (1.23-fold) and a greater increase after long (60 min) isoflurane anesthesia (2.47-fold). Finally, they reported a 39% increase in ^18^F-FDG uptake in BAT of animals stimulated by cold exposure.

Fluorescence imaging is based on the detection of light coming from exogenous fluorochromes (dyes or genetically engineered fluorescent proteins) excited with light sources (laser or lamps). The photons escaping from the sample surface are generally detected by a charge CCD with high quantum efficiency. Excitation and emission wavelengths suitable for *in vivo* investigations are in the red-near infrared region (650–800 nm) where the biological tissues are optically thin. Outside this “transparency window” the substantial absorption of oxy- and deoxy-hemoglobin, melanin, water and fat reduces the light signal. Fluorescence imaging is a very simple, safe, and cost-effective technique, which has been implemented to obtain tomographic 3D reconstructions of the light sources inside the body (Beckmann et al., 2007).

A pure optical approach to the detection of BAT was recently reported by Rice et al. (2015) who administered mice with a micellar formulation of commercially available deep-red fluorescent probe (SRFluor680). They showed an extensive uptake of the fluorescent probe in iBAT, as clearly visible in **Figure 4B**. The results were confirmed by ^18^F-FDG PET imaging and *ex vivo* examinations. They explained the results by an irreversible translocation of the lipophilic fluorescent probe from the micelle nanocarrier to the adipocytes within the BAT. The authors suggest that combining optical methods with FDG/PET could constitute a path toward a new molecular imaging paradigm allowing non-invasive visualization of BAT mass and BAT metabolism in living subjects.

## Conclusion

Magnetic Resonance Imaging, CT, and PET represent a panel of imaging techniques which are instrumental for the *in vivo* detection and quantification of fat tissues in cross sectional and longitudinal studies. Several qualitative and quantitative morphological and functional data can be extracted from images, allowing for the investigation of fat tissue metabolism and drug response. Techniques based on MRI and MRS are considered the most comprehensive tools for quantification of fat in living organisms. MRI based techniques allow to investigate the anatomical distribution of adipose tissues, the presence of ectopic fat, and also chemical and functional state of fat deposits with high space resolution. MicroCT can discriminate fat tissue from remaining soft tissue in small laboratory animals with similar space resolution. PET suffers from limited space resolution (around 1 mm), from the need for expensive radiotracers and controlled environment, but it has been proven to be extremely sensitive in studies of BAT activation. Indeed MRI, CT and PET should be regarded as a set of complementary techniques. Much effort is currently in progress toward multimodal imaging approaches: hybrid instruments combining PET and MRI, or PET and CT, have been developed also for small animal imaging to overcome the limitations of individual techniques. MRI, CT, and PET are used in both clinical and preclinical fields, thereby enhancing the translational value of findings in experimental animals. A limitation of these techniques is the high cost of instrumentation and maintenance, and the need for specialized personnel. Accordingly, large studies employing tens or hundreds of animals are cumbersome. However, microCT, PET and, especially, MRI are non-invasive and allow for longitudinal studies where a reduced number of animals is sufficient in order to obtain statistically significant results. Recently other imaging techniques (CLI and fluorescence imaging) have been applied to these topics, but still await full validation.

## Author Contributions

All authors listed, have made substantial, direct and intellectual contribution to the work, and approved it for publication.

## Conflict of Interest Statement

FM is employedin Bruker Italia s.r.l., and the other authors declare that the research was conducted in the absence of any commercial or financial relationships that could be construed as a potential conflict of interest.
